# Near-ideal electromechanical coupling in textured piezoelectric ceramics

**DOI:** 10.1038/s41467-022-31165-y

**Published:** 2022-06-22

**Authors:** Yongke Yan, Liwei D. Geng, Hairui Liu, Haoyang Leng, Xiaotian Li, Yu U. Wang, Shashank Priya

**Affiliations:** 1 Department of Materials Science and Engineering, Pennsylvania State University, University Park, PA 16802 USA; 2 Department of Materials Science and Engineering, Michigan Technological University, Houghton, MI 49931 USA

**Keywords:** Actuators, Electronic devices

## Abstract

Electromechanical coupling factor, *k*, of piezoelectric materials determines the conversion efficiency of mechanical to electrical energy or electrical to mechanical energy. Here, we provide an fundamental approach to design piezoelectric materials that provide near-ideal magnitude of *k*, via exploiting the electrocrystalline anisotropy through fabrication of grain-oriented or textured ceramics. Coupled phase field simulation and experimental investigation on <001> textured Pb(Mg_1/3_Nb_2/3_)O_3_-Pb(Zr,Ti)O_3_ ceramics illustrate that *k* can reach same magnitude as that for a single crystal, far beyond the average value of traditional ceramics. To provide atomistic-scale understanding of our approach, we employ a theoretical model to determine the physical origin of *k* in perovskite ferroelectrics and find that strong covalent bonding between B-site cation and oxygen via *d*-*p* hybridization contributes most towards the magnitude of *k*. This demonstration of near-ideal *k* value in textured ceramics will have tremendous impact on design of ultra-wide bandwidth, high efficiency, high power density, and high stability piezoelectric devices.

## Abstract (web summary)

“*k*” is the electromechanical coupling factor, and determines the efficiency of conversion between electrical and mechanical energy or vice-versa in piezoelectric materials. Here, Yan et al. present an optimization strategy for “*k*” via texturing and covalency tuning that results in “*k*” values comparable to that of single crystals.

## Introduction

Piezoelectric materials enable the electromechanical conversion between electrical energy and mechanical energy and vice-versa. They are widely utilized in sensors, actuators, transducers, imaging devices, and energy harvesters^1,2^. The electromechanical coupling factor, *k*, quantifies the effectiveness of piezoelectric material in providing conversion between electrical energy and mechanical energy and vice-versa. The parameter, *k*^*2*^, reflects the ratio of stored mechanical energy to input electrical energy, or the ratio of stored electrical energy to input mechanical energy, given as: *k*^2^ = stored mechanical energy/input electrical energy, or, *k*^2^ = stored electrical energy/input mechanical energy^3^. The piezoelectric materials with high *k* will provide high maximum achievable bandwidth and maximum powder density with high efficiency, and hence the *k* is one of the most important parameters for piezoelectric transduction devices^3^.

As another particular benefit, the design of the piezoelectric materials with high *k* can provide an alternative approach for increasing the piezoelectric coefficient *d* according to the relation: $d=k\sqrt{s\cdot \varepsilon }$, where *s* is the elastic compliance and *ε* is the dielectric permittivity^3^. This approach overcomes several bottlenecks of the traditional approach via composition design. With the traditional approach, the enhancement of the piezoelectric response *d* is achieved by increasing the dielectric permittivity *ε* based on the expression, *d* = 2*QP*_*s*_*ε*, where *P*_s_ is the spontaneous polarization, *Q* is the electrostrictive coefficient and *ε* is the dielectric permittivity^4^. The increase of *ε* is realized via flattening the free energy landscape with respect to the polarization (lowering the energy barrier for ferroelectric polarization rotation), specifically, by means of designing composition-induced multiphase coexistence (such as morphotropic phase boundary and polymorphic phase transition)^5,6^ or engineering local compositional structures (such as nanoscale short-range ordering^7^ and local structural heterogeneity^8,9^). The bottlenecks associated with this traditional approach include: (1) Enhanced piezoelectric property *d* is obtained at the cost of their temperature stability (lower depolarization temperature *T*_d_ or low Curie temperature *T*_c_), which follows a trend given as: *d* ∝ 1/*T*^1,10,11^; (2) The increase of *ε* decreases the piezoelectric voltage coefficient *g*, resulting in reduced sensitivity as a piezoelectric sensor^12^; (3) This approach is not effective in improving the energy density of piezoelectric materials, characterized by *d*·*g*^13^. For example, even though the *d*_33_ of Sm-doped Pb(Mg_1/3_Nb_2/3_)O_3_-PbTiO_3_ (abbreviated as PMN-PT) random ceramics can reach 1500 pC N^−1^ by increasing the *ε*_33_ to 13,000, the *d*_33_·*g*_33_ is limited to 19.8 × 10^−12^ m^2 ^N^−1^, which is only 1/3 of the value of textured Pb(Mg_1/3_Nb_2/3_)O_3_-Pb(Zr,Ti)O_3_ (abbreviated as PMN-PZT) ceramics^9,13^; (4) The increase of *d* cannot give rise to a significant increment of *k*, which is one of the most important factors for piezoelectric devices as mentioned above; (5) Increasing the dielectric permittivity *ε* by flattening the free energy landscape generally reduces the coercive field *E*_c_, which weakens the electric field stability (depoling) and limits the use of material in high power application. Here we show that these challenges can be overcome by designing piezoelectric materials with high *k*.

In about 75 years of piezoelectric materials history, high *k* of over 0.9 has been only observed in domain engineered single crystals, rather than polycrystalline ceramics. For example, [001]-oriented PMN-PT and Pb(Zn_1/3_Nb_2/3_)O_3_-PbTiO_3_ (abbreviated as PZN-PT) single crystals possess *k*_33_ of over 0.9^14^, far beyond the value of ~0.7 of piezoelectric ceramics^1^. However, the broad application of single crystals is limited by the cost, dimension, and composition inhomogeneity. Synthesis of textured polycrystalline ceramics could offer a better performance/cost ratio, potentially achieving piezoelectric properties close to those of single crystals but at a low cost like that of polycrystalline ceramics^12,13,15–17^. Prior research has shown that <001> oriented textured piezoelectrics can have higher *k* than those of their random counterparts^13,18,19^. This provides us initial direction towards addressing several questions: what is the maximum value of *k* that can be achieved in textured ceramic and is it possible to obtain the same or even higher *k* in textured ceramics than the values in their single-crystal counterparts? Does the neighboring grain correlation in textured ceramic limit the maximum achievable *k*? Does this approach of increasing *k* by microstructure texturing show the advantages over the traditional approach of increasing dielectric permittivity *ε* by composition design as mentioned above? In order to answer these questions, we carried out phase-field simulation to investigate the effects of crystallographic orientation and grain boundary in textured ceramics on the electromechanical coupling factor *k*. Results from the simulation were experimentally verified to confirm the increase in *k* in highly <001> textured PMN-PZT ceramics. A theoretical model is developed to gain an understanding of the physical origin of electromechanical coupling in perovskite ferroelectrics and determine the key correlations.

## Results

### Phase-field simulation of ultrahigh electromechanical coupling in textured piezoelectric ceramics

**Fig. 1:**
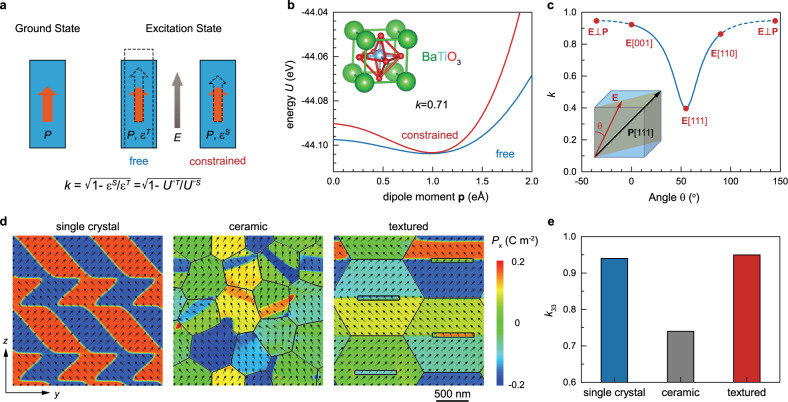
Electromechanical coupling of piezoelectric materials. **a** Definition of electromechanical coupling factor, *k*. The parameter *k* characterizes the conversion between electrical energy and mechanical energy, which is related to two different excitation states of polarization in ferroelectric materials, namely, the free and the constrained. **b** First-principles calculated energy profiles of BaTiO_3_ with free and constrained unit cell. **c** Simulated *k* for the rhombohedral PMN-PT single crystal under the electric field with various orientations. **d** Domain structures, and **e** corresponding longitudinal electromechanical coupling factor *k*_33_ of single crystal, random ceramic and textured ceramic after the electrical poling along [001] direction.

The electromechanical coupling factor *k* characterizes the conversion between electrical energy and mechanical energy and is related to two different excitation states of polarization in ferroelectric materials, namely, the free and the constrained. As illustrated in Fig. 1a, larger polarizations can be induced by the external electric field, but the polarization increment could be different in the two excitation cases. Constraining the sample often results in a smaller polarization change and thus gives rise to a lower dielectric permittivity, *ε*^*S*^, in comparison with the free state where the permittivity is *ε*^*T*^. The electromechanical coupling factor *k* can be determined by the permittivity ratio of the two cases, i.e., $k=\sqrt{1-{\varepsilon }^{S}/{\varepsilon }^{T}}$. The total permittivity in ferroelectrics may usually be separated into three parts, electronic, dipole, and domain wall motion. The electronic contribution arises from the displacement of the electron shell relative to a nucleus and is often negligible in ferroelectric materials with high permittivity. The contribution of domain wall motion plays a less important role in poled ferroelectrics or at a higher frequency, especially for polycrystal ceramics where grain boundaries may further suppress the motion. Therefore, the permittivity is mainly contributed by the dipole part, i.e., polarization extension and rotation. From the energy point of view, the permittivity is determined from the curvature of energy profile *U*(*p*) around the spontaneous polarization, and hence, the factor *k* is related to the curvature ratio of the two cases, i.e., $k=\sqrt{1-U\hbox{''}^{T}/U\hbox{''}^{S}}$. As an example, the electromechanical coupling factor for the typical perovskite ferroelectrics, BaTiO_3_, is calculated using the density-functional theory (DFT) (Fig. 1b). As expected, the energy profile of BaTiO_3_ with constrained unit cell exhibits a larger curvature around the spontaneous dipole moment, because of the exerted constraint that usually leads to more difficult ionic displacement. It is worth noting that, *k* is not determined by the individual permittivity or energy profile, i.e., large permittivity is not the necessary condition for high *k*.

Giant electromechanical coupling (*k* > 0.9) was observed for relaxor-PT single crystals, and the magnitude of *k* is orientation-dependent^20^. Figure 1c shows the simulated *k* for the rhombohedral PMN-PT single crystal under the electric field with various orientations. The corresponding simulated permittivity *ε*^*T*^ and *ε*^*S*^ are shown in Supplementary Fig. 1. Based on the microscopic model, the magnitude of *k* depends on the competition between chemical energy and elastic energy. Since both energies are polarization-dependent and anisotropic, *k* must be also anisotropic and thus depend on the electric field orientation. Because of the strong strain constraint, the change of *ε*^*S*^ is almost negligible in comparison with that of *ε*^*T*^. Therefore, the anisotropic behavior of *k* will be mainly determined by *ε*^*T*^. Since the polarization of PMN-PT behaves as a rotator rather than an extender^21^, the permittivity and thus *k* will be higher if the angle between electric field and polarization is larger. As shown in Fig. 1c, the highest *k* occurs when the electric field is perpendicular to polarization, while the lowest *k* occurs when the electric field is parallel with polarization, which suggests that *k*_15_ mode possesses the largest value of *k* in rhombohedral relaxor-PT single crystals^22^. However, upon electrical poling, only the values of *k* with *θ* ∈ [0,90°] are allowed, indicated by the solid blue line in Fig. 1c. Among those values, the highest *k* occurs when the electric field is along [001] direction, i.e., [001]-poled single crystal can exhibit the highest *k*_33_. In polycrystal ceramics, however, since the grain orientations are randomly distributed, the highest *k*_33_ can’t be obtained as in single crystals. Nevertheless, the [001]-textured polycrystal can help to realize the highest *k*_33_ in ceramics. For a [001]-textured polycrystal, all grains are oriented in [001] crystallographic axis while the other crystallographic axes are completely random. Even though the [001]-textured polycrystal can possess the highest *k*_33_ in ceramics, can it be as large as that of a single crystal?

**Fig. 2:**
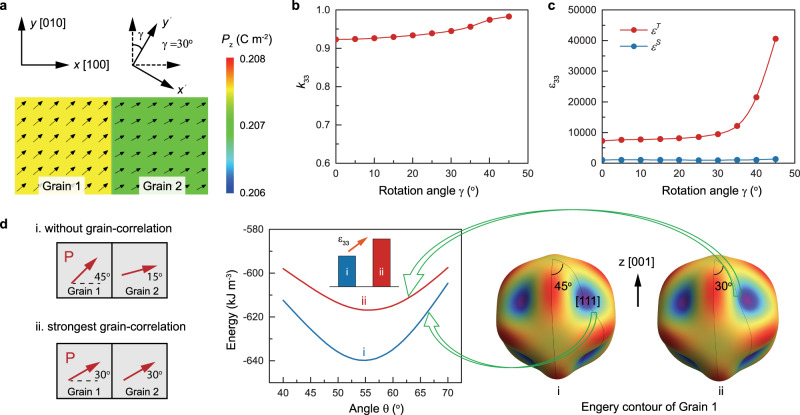
Grain-correlation effect on k33 determined by phase-field simulation and Landau theory. **a** Polarization structure for a two-grain ferroelectric system, where the grain orientations are illustrated by the axes. **b** Simulated *k*_33_ as a function of rotation angle *γ* between the orientations of two grains. **c** Simulated permittivity *ε*^*T*^ and *ε*^*S*^ as a function of rotation angle *γ*, where the electric field is applied along [001] direction. **d** Illustration of the grain-correlation effect on the permittivity based on the Landau theory: grain-correlation will make the polarization rotation path deviate from its equilibrium point and hence flattens the energy profile, which increases the permittivity *ε*^*T*^ and eventually enhances *k*_33_.

For highly textured ceramics, the main difference from single crystal is the correlation between neighboring grains due to the existence of grain boundaries. Figure 2 illustrates the grain-correlation effect on *k*_33_ by performing phase-field simulations on a two-grain system. The polarization structure of the two-grain ferroelectric system is shown in Fig. 2a. The simulated *k*_33_ and *ε*_33_ are shown in Fig. 2b, c, respectively. *k*_33_ is found to be slightly enhanced due to the correlation between the two grains, which is mainly attributed to the enhancement of permittivity *ε*^*T*^. The grain-correlation effect on the permittivity *ε*^*T*^ is illustrated based on the Landau theory as shown in Fig. 2d. The grain correlation will make the polarization rotation path deviate from its equilibrium point and hence flattens the energy profile, which increases the permittivity *ε*^*T*^ and eventually enhances *k*_33_. The larger the grain-correlation effect, the higher the electromechanical coupling. Thus, based on the above analysis, a large *k*_33_ that is even higher than that of single crystals can be achieved in textured polycrystal ceramics. To further examine this possibility, phase-field simulations are performed on the single crystal, random ceramic, and [001]-textured ceramic systems. Fig. 1d shows the domain configurations as well as domain walls for the three systems after the electrical poling along [001] direction, where the black arrows represent the in-plane (*y-z*) components of polarization and the color contours represent the out-of-plane (*x*) component. Upon electrical poling, there are four equivalent polarization directions in the rhombohedral phase. For a single crystal, the four equivalent domains that are separated by 71^o^ and 109^o^ domain walls are well arranged to form the typical “herringbone” pattern. For random ceramic, the regular domain patterns only exist in individual grains while the overall ceramic doesn’t show a well-arranged domain structure. For textured ceramic, whose grain size is larger than the random ceramic, the well-aligned stripe domain pattern is formed, which is analogous to stripe domains separated by 109^o^ domain walls in a single crystal. The emergence of single-crystal-like domain structures originates from the fixed [001] crystallographic axis within grains with a high Lotgering factor. But this condition is relaxed for the other two axes whose orientations are randomly distributed, resulting in electrical and elastic correlations that can’t be neglected between neighboring grains. This correlation drives the emergence of stripe-like domain patterns with non-109^o^ domain walls. These stripe-like domain structures are not present in the randomly oriented polycrystalline ceramics. Since domain walls may also play a role in the electromechanical coupling, we have examined the domain wall effect on *k* by considering different numbers of domain walls. The simulated results are shown in Supplementary Fig. 2, which reveal that the existence of domain walls can slightly enhance the electromechanical coupling. The corresponding simulated values of *k*_33_ are shown in Fig. 1e. As expected, the random ceramic exhibits the smallest *k*_33_, while the [001]-textured ceramic possesses a large *k*_33_ that is comparable to (and even slightly higher than) that of the single crystal. These findings suggest that ultrahigh electromechanical coupling factor *k*_33_ can be achieved in ceramics by following the proposed texturing design.

### Ultrahigh electromechanical coupling in textured PMN-PZT ceramics

**Fig. 3:**
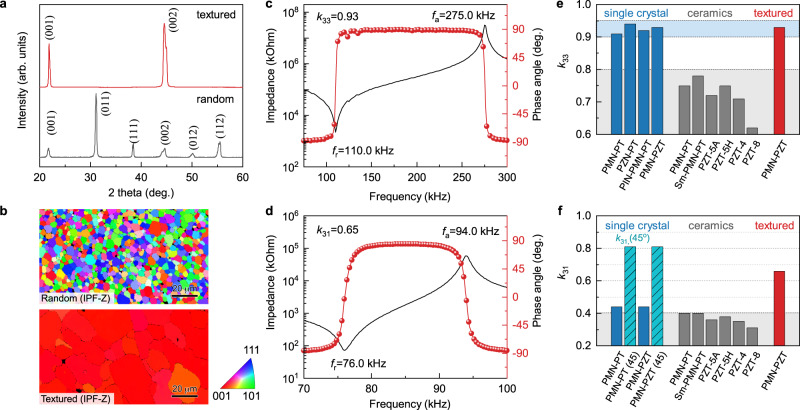
Textured PMN-PZT ceramics. **a** XRD patterns of random and textured PMN-PZT ceramics. **b** EBSD images of random and <001> textured PMN-PZT ceramics. **c** Impedance spectra of <001> textured PMN-PZT ceramics in longitudinal 33 mode, the electromechanical coupling factor, *k*_33_, is as high as 0.93. **d** Impedance spectra of <001> textured PMN-PZT ceramics in transverse 31 mode, the electromechanical coupling factor, *k*_31_, is as high as 0.65. **e** Comparison of *k*_33_ among [001] oriented single crystals, random ceramics and <001> textured ceramics. **f** Comparison of *k*_31_ among [001] oriented single crystals, random ceramics and <001> textured ceramics. Here *k*_31_(45^o^) is the *k*_31_ of [001] oriented single crystal sample with 45^o^ cut, where the orientations of the sides are [110], [$\bar{1}10$] and [001], respectively. The references for *k*_33_ and *k*_31_ data for single crystals and ceramics in **e**, **f** can be found in Supplementary Note 1.

To experimentally verify the prediction that the <001>-textured ceramic can possess a large *k* that is comparable to the value of the single crystal, highly <001>-textured PMN-PZT ceramic was prepared by templated grain growth technique with a different volume percentage of BaTiO_3_ templates. Figure 3a shows the X-ray diffraction patterns for random PMN-PZT ceramic with 0 vol% BaTiO_3_ templates and textured PMN-PZT ceramic with 3 vol% BaTiO_3_ templates, respectively. Both samples exhibit a perovskite phase, while textured ceramic shows a remarkable enhancement in the intensities of the {001} diffraction peaks compared to random ceramic. The Lotgering factor of the textured sample is over 98%, indicating a strong [001] preferred grain orientation. Figure 3b shows the grain orientation of random and textured PMN-PZT ceramics via inverse pole figure (IPF) maps measured by the SEM-EBSD technique along thickness (Z) direction (The IPF-X and IPF-Y maps are shown in Supplementary Fig. 3). In order to evaluate the electromechanical coupling, longitudinal 33 mode and transverse 31 mode samples with dimensions according to IEEE standards were prepared, and their impedance spectra are shown in Fig. 3c, d. The *k*_33_ and *k*_31_ of <001> textured PMN-PZT are surprisingly as high as 0.93 and 0.65, respectively. Figure 3e lists the *k*_33_ of representative [001] oriented single crystals and polycrystalline random ceramics. The *k*_33_ of <001> single crystals are in the range of 0.90–0.94, while the *k*_33_ of random ceramics are limited to below 0.80. The *k*_33_ of <001> textured PMN-PZT is the same as the value of PMN-PZT single crystal counterpart. Figure 3f lists the *k*_31_ of representative [001] oriented single crystals and polycrystalline random ceramics. In general, the *k*_31_ of random ceramics are in the range of 0.30–0.40. The *k*_31_ and *k*_31_(45^o^) of [001] oriented single crystals are about 0.43 and 0.80, respectively. Here *k*_31_(45^o^) is the *k*_31_ of [001] oriented single crystal sample with 45^o^ cut, where the orientations of the sides are [110], [$\bar{1}10$] and [001], respectively. The *k*_31_ in textured PMN-PZT ceramic is 0.65, which is slightly higher than the average value of *k*_31_ and *k*_31_(45^o^) of [001] oriented single crystals. This characteristic is due to the distribution of grain orientation in <001> textured ceramics, where the [001]-orientation of grains in textured samples are well aligned along the thickness direction (*z*, out of casting plane) but the [100] and [010] orientations of grains in textured samples are randomly distributed in the casting plane, which is related to the fact that unidirectional shear force was used for aligning the templates. Based on these observations, it can be suggested that the *k*_,_ either *k*_31_ or *k*_33_, in textured ceramic is solely dependent on the texture direction and texture degree, and is not limited by the existence of the grain boundary. The <001>-textured ceramic can possess a large *k*_33_ that is comparable to those of the <001>-oriented single crystals, which verifies the prediction in Figs. 1 and 2.

**Fig. 4:**
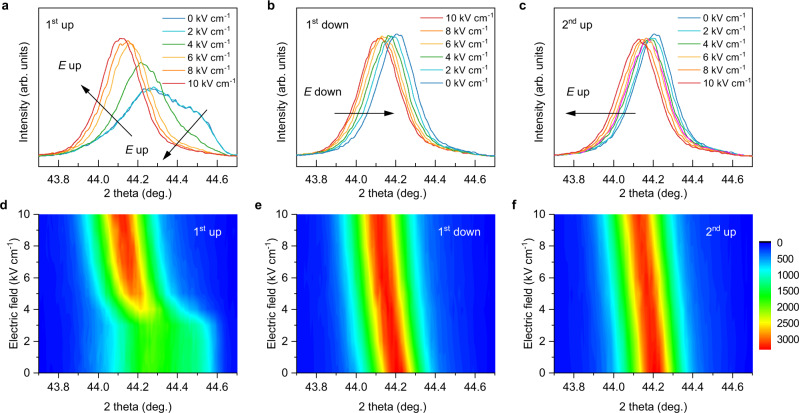
Electric-field-induced structure evolution. In-situ electric field XRD patterns of textured PMN-PZT-3BT ceramics. **a** Increase electric field on unpoled sample, denoted as 1st up. **b** Then decrease electric field to 0, denoted as 1st down. **c** Then increase the electric field again, denoted as 2nd up. **d** Contour plots of in-situ XRD data from (**a**). **e** Contour plots of in-situ XRD data from (**b**). **f** Contour plots of in-situ XRD data from (**c**).

The same *k* of [001]-oriented single crystal and <001>-textured ceramics suggests that they may possess similar domain configurations. It is well known that the ability of domains to switch in ferroelectric polycrystals depends critically on the crystallographic symmetry of the ferroelectric phase^23^. The domains in random polycrystal ceramics that are either tetragonal or rhombohedral are difficult to switch due to the constrain by the differently oriented neighboring grains. <001> texture is requisite for non-180^o^ domain switching in tetragonal phase^23^. Figure 4 shows the in-situ electric field XRD patterns of <001>-textured PMN-PZT-3BT ceramics. It can be observed that the unpoled textured sample has MPB composition with the coexistence of rhombohedral and tetragonal phases (Fig. 4a), characterized by peak splitting near 44^o^. With increasing the electric field (1st up), the high angle peak (*a*-axis) is merged into the low angle peak (*c*-axis) and becomes a single peak, indicating that the *a*-domains in the tetragonal phase can be fully switched to *c*-domains. This new domain structure is very stable during the removal of the electric field (1st down, Fig. 4b) and application of electric field (2nd up, Fig. 4c). Based on these observations, it can be suggested that the non-180^o^ domains in <001> textured ceramics are switchable, further confirming that <001>-textured ceramics could have the same electromechanical coupling factor *k* as [001]-oriented single crystal.

### Temperature dependence of dielectric and piezoelectric properties of textured PMN-PZT ceramics

One of the main advantages of texturing approach over the composition approach (increasing the dielectric permittivity *ε* via composition and phase structure design) is that maximizing *k* via orientation control will provide better comprehensive piezoelectric properties and temperature stability. The temperature dependence of *k*_33_ and ${\varepsilon }_{33}^{T}$ has also been illustrated by phase-field simulation as shown in Supplementary Fig. 4. The ${\varepsilon }_{33}^{T}$ shows strong temperature dependence and increases significantly as the temperature approaches the phase transition due to the flattening of the free energy landscape. However, according to $k=\sqrt{1-{\varepsilon }^{S}/{\varepsilon }^{T}}$, such a significant increase of *ε*^*T*^ will not result in the large variation of *k*, especially for the piezoelectric material with high *k*, where the change of *ε*^*S*^ is almost negligible in comparison with that of *ε*^*T*^ under the strong strain constraint.

**Fig. 5:**
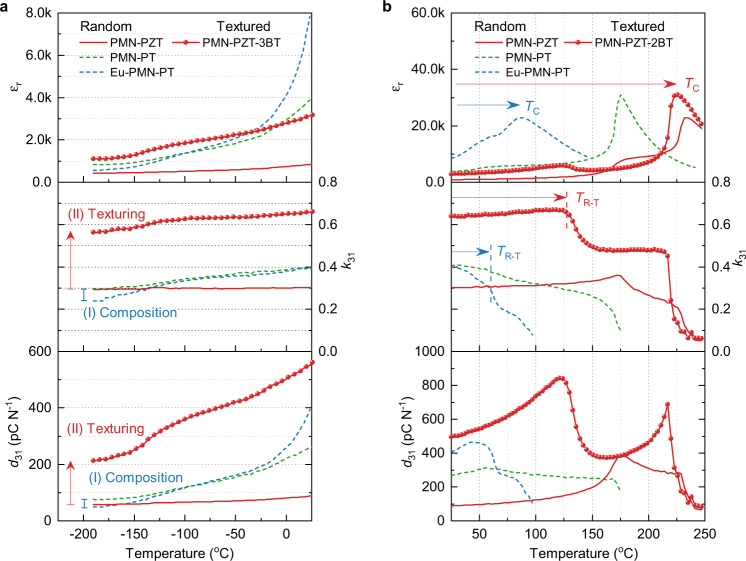
Temperature dependence of the dielectric, piezoelectric, and electromechanical properties of textured PMN-PZT ceramics. **a** Properties measured in a low-temperature range from −190 °C to room temperature, **b** Properties measured in a high-temperature range from room temperature to 250 °C. The texturing approach provides much larger increments of *d* and *k* as well as a wider temperature range (as indicated by higher rhombohedral to tetragonal ferroelectric-ferroelectric phase transition *T*_R-T_ and ferroelectric-paraelectric phase transition *T*_C_) than the traditional composition approach, as indicated by the vertical and horizontal arrows.

Figure 5 compares the experimental data on temperature-dependent dielectric, piezoelectric and electromechanical properties of piezoelectric materials designed by these two approaches at both low-temperature and high-temperature ranges. For the composition approach, PMN-PT system was chosen because its phase diagram has been well established. Specifically, PMN-35PT and 2.5 mol% Eu_2_O_3_ doped PMN-28PT were selected as undoped and doped PMN-PT, respectively. Both these two compositions are near the MPB and show the peak values of piezoelectric coefficient *d*. As shown in Fig. 5a, at room temperature, the dielectric constant *ε*_r_ of Eu-doped PMN-PT is significantly higher than that of undoped PMN-PT, leading to higher piezoelectric coefficient *d*_31_. However, both the dielectric constant *ε*_r_ and piezoelectric coefficient *d*_31_ of Eu-doped PMN-PT sample show much stronger temperature dependence and even have lower values than undoped PMN-PT at cryogenic temperatures. At low temperatures, the dielectric constant *ε*_r_ and the piezoelectric coefficient of *d*_31_ for undoped and doped samples are gradually merged together due to the diminishing contribution of polar nano regions (PNRs)^24^ or local heterogeneity induced by Eu rare earth dopant^9^.

For the microstructural texturing approach, the electromechanical coupling factor *k* can get significantly improved, leading to a large increment of *d*. Meanwhile, *d* exhibits much better temperature stability than the compositional approach because *k* has much less temperature sensitivity than *ε*_r_. An extraordinary example here is textured PMN-PZT. At room temperature, texturing increases *k*_31_ of PMN-PZT from 0.3 to 0.65, correspondingly, significantly increases *d*_31_ from 89 pC N^−1^ to 568 pC N^−1^, *d*_33_ from 230 to 1470 pC N^−1^, *g*_33_ from 28 × 10^−3^ V m N^−1^ to 50 × 10^−3^ V m N^−1^, and *d*_33_·*g*_33_ from 6.5 × 10^−12^ m^2^ N^−1^ to 73 × 10^−12^ m^2^ N^−1^, respectively. At low temperatures, the increments of *d*_31_ and *k*_31_ via texturing approach are also much larger than the composition approach as shown in Fig. 5a. Similar to the low temperature, texturing remarkably increases the *k*_31_ of PMN-PZT and increases *d*_31_ of PMN-PZT in high-temperature range as shown in Fig. 5b. The textured PMN-PZT shows higher *k*_31_ and *d*_31_ and a wider operating temperature range than PMN-PT. For random ceramic, achieving high *d* at room temperature always sacrifices the temperature stability, characterized by low ferroelectric-ferroelectric phase transition (*T*_R-T_) and ferroelectric-paraelectric phase transition *T*_C_. It should be mentioned here that the grain orientation or texturing will not affect the phase transition temperature. Here, the slight decrease in phase transition temperatures in textured PMN-PZT is due to the low Curie temperature of BaTiO_3_ templates (130 °C). This can be solved by using a homogeneous PMN-PZT template that has the same composition as that of the matrix. Furthermore, as shown in Supplementary Fig. 5, although Eu doping significantly increases the dielectric permittivity *ε* and piezoelectric properties *d*, it dramatically reduces the coercive field *E*_c_, which weakens the electric field stability (depoling) and limits the use of the material in high power application. On the contrary, texturing has much less impact on the coercive field *E*_c_. In addition, as shown in Supplementary Fig. 6, unlike Eu doping that increases the dielectric loss, tan*δ*, of PMN-PT random ceramics at room temperature from 1.8% to 3.4%, the [001]-texturing significantly reduces tan*δ* of PMN-PZT random ceramics at the room temperature from 2.3% to 1.1%. A similar trend also has been observed in [001]-textured PMN-PT ceramics where tan*δ* at the room temperature reduces from 2.0% to 0.6%^18^. Overall, as summarized in Supplementary Fig. 7, our approach based upon microstructural texturing provides high *k* (wide bandwidth and high energy transduction efficiency), high *d* (large strain), large *d*·*g* (high transduction energy density), and high *g* (large sensitivity), low loss without significantly sacrificing temperature and electric-field stability, which overcomes the aforementioned bottlenecks presented by the traditional approach (increasing the dielectric permittivity *ε*) based on composition design (doping and solid solution).

### Origin of electromechanical coupling in ferroelectrics

**Fig. 6:**
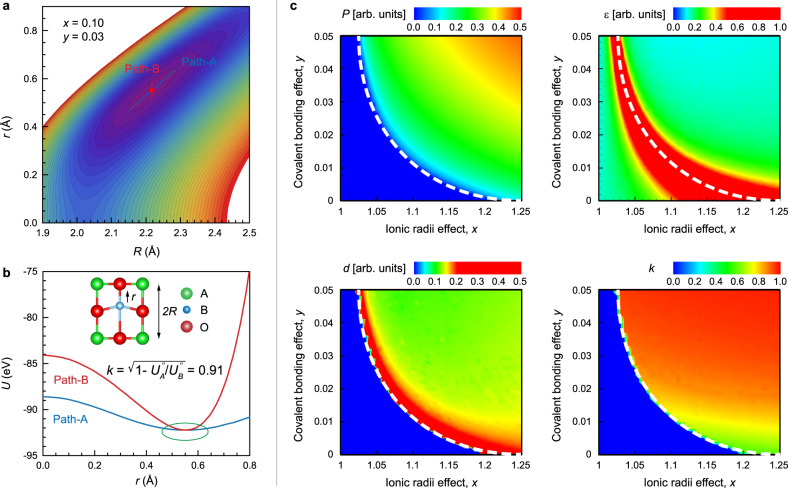
Origin of electromechanical coupling in ferroelectrics explained using a theoretical model. **a** Energy contour for a ferroelectric state as a function of ionic displacement *r* and lattice deformation *R*. **b** Energy profiles along Path-A and Path-B. **c** Color contours of polarization *P*, permittivity *ε*, piezoelectricity *d*, and electromechanical coupling factor *k*, as a function of ionic radii effect *x* and covalent bonding effect *y*. *x ≡ ρ*_*A*_ /*ρ*_*B*_ implies the ionic radius ratio between A and B ions, while *y ≡ λ*_*Bc*_ /*λ*_*B*_ characterizes the relative covalent bonding strength of the B-O bond. The white dash line depicts the paraelectric-ferroelectric phase transition boundary.

Considering the importance of electromechanical coupling, it is crucial to design new ferroelectric materials with a very high *k*. To do so, the factors that impact *k* should be examined. Herein, we employ a theoretical model to understand the origin of electromechanical coupling in perovskite ferroelectrics and explore the key factors that contribute most to *k*. The modeling details can be found in Supplementary Note 2. From the energetic point of view, off-center ionic displacements can lead to a lower energy for ferroelectric phases. Figure 6a contours present the energy *U* as a function of ionic displacement *r* and lattice deformation *R* for a ferroelectric state. Usually, the ionic displacement (polarization) and lattice deformation (strain) are coupled, for example, a larger displacement can also induce a larger deformation. Therefore, if the deformation is conserved (Path-B), the energy *U*_*A*_(*r*) will be higher than the energy *U*_*B*_(*r*) with free deformation (Path-A). The electromechanical coupling factor *k* is related to the ratio of the two energy curvatures, as shown in Fig. 6b.

The origin of ferroelectricity was first discussed by Megaw^25^ in 1952 and then explained computationally by Cohen^26^ 40 years later. In perovskite ferroelectrics, the short-range repulsive forces that are mainly caused by the Pauli exclusion principle favor the ideal structure while the long-range Coulomb forces (Madelung) favor off-centering ionic displacements, the ferroelectricity is hence determined by the competition between short-range and long-range interactions. The covalent bonding between B-cation and oxygen via *d-p* hybridization can reduce the short-range repulsion and thus allow off-centering displacements to create ferroelectricity^26^. In fact, besides B-O hybridization, the ionic radii can also impact the energy competition and hence the ferroelectricity. Fig. 6c shows the mapping of ferroelectricity (*P*) as a function of ionic radius ratio *x* and relative covalent bonding strength *y*. If the ionic radius of B is comparable to that of A, it is very hard to induce the ferroelectricity even for a strong B-O covalent bonding. However, if the ionic radius of B is much smaller than that of A, the ferroelectricity still can be induced even without the B-O covalent bonding, where the small B ion displaces easily within the large cage of O ions. Based on our theoretical results, large spontaneous polarization can be achieved by choosing a large A ion or small B ion with a strong B-O hybridization.

Since ionic radii and covalent bonding play an important role in ferroelectricity, their impact on electromechanical coupling is also examined. The color contours of permittivity *ε*, piezoelectricity *d*, and electromechanical coupling factor *k*, as a function of ionic radius ratio *x* and relative covalent bonding strength *y*, are shown in Fig. 6c. As expected, large permittivity *ε* appears around the phase transition boundary where the energy profile of *U*(*r*) will be more flattened, and no obvious polarization dependency can be found. Similarly, large piezoelectricity *d* also appears near the phase transition boundary where the energy profile of *U*(*R*) will be more flattened, but it depends on the polarization. However, the electromechanical coupling factor *k* exhibits a different behavior: large *k* does not require a large permittivity or piezoelectricity. Since the permittivity or piezoelectricity is related to path-A while the electromechanical coupling is related to both Path-A and Path-B, there is no direct relationship between them. The theoretical model reveals that larger *k* results from larger ionic radius of A or smaller ionic radius of B as well as stronger B-O covalent bonding. However, very large *k* (>0.9) prefers the strong covalent bonding effect rather than the ionic effect. These findings suggest an optimal condition for perovskite ferroelectrics that simultaneously possess a large permittivity, large piezoelectricity, and a high electromechanical coupling factor: strong B-O covalent bonding and close to the phase transition boundary. Similar studies based on experimental measurements to analyze the effects of A-site or B-site ions on the electromechanical coupling factor have been reported by Yamashita^27–30^.

In summary, we provide a breakthrough in the design of piezoelectric ceramics that achieves a near-ideal magnitude of *k* via grain orientation/texturing. Through phase-field simulation and experimental verification by textured PMN-PZT, it was found that textured ceramics can achieve the same *k* as a single crystal. The *k*_33_ of highly <001> textured PMN-PZT is measured to be 0.93, which closes the gap between the ceramic and single crystal. In addition, increasing *k* via texturing provides an alternative approach of increasing the piezoelectricity, which overcomes the bottlenecks of the traditional approach via composition design. Further, we employed a theoretical model to understand the physical origin of *k* in perovskite ferroelectrics and found that strong covalent bonding between B-cation and oxygen via *d*-*p* hybridization contributes most to *k*. These findings provide a novel design strategy to develop the next generation of high-performance piezoelectric materials with ultrahigh piezoelectricity and low cost, to fulfill the demands for ultra-wide bandwidth, high efficiency, high power density, and high stability piezoelectric devices.

## Methods

### Synthesis of <001> textured PMN-PZT ceramics

The piezoelectric textured ceramics were synthesized by templated grain growth process as illustrated in Supplementary Fig. 8. The 0.40Pb(Mg_1/3_Nb_2/3_)O_3_-0.25PbZrO_3_-0.35PbTiO_3_ (PMN-PZT) matrix powder was synthesized by the conventional solid-state reaction method. A mixture of PbO (99.9%, Sigma Aldrich, USA), MgNb_2_O_6_ (99.9%, Alfa Aesar, USA), ZrO_2_ (99%, Sigma Aldrich, USA) and TiO_2_ (99.9%, Sigma Aldrich, USA) was ball-milled in ethanol for 24 h using ZrO_2_ milling media (Tosoh USA). After the drying process, the ball-milled mixture was dried and calcined at 750 °C for 2 h. Calcined powder was ball-milled again with 1.5 wt% excess PbO for 24 h. The templates for texturing PMN-PZT ceramics are plate-like [001]_PC_ BaTiO_3_ (BT) microcrystals. To synthesize the [001]_PC_ BT templates, three steps were involved. Firstly, Bi_4_Ti_3_O_12_ platelets were synthesized by reacting Bi_2_O_3_ with TiO_2_ powders in NaCl and KCl molten salts at 1050 °C for 1 h. Next, BaBi_4_Ti_4_O_15_ platelets were synthesized by reacting Bi_4_Ti_3_O_12_ with TiO_2_ and BaCO_3_ in BaCl_2_/KCl molten salts at 1050 °C for 3 h. Finally, [001]_PC_ BT platelets were obtained by topochemical reaction between BaBi_4_Ti_4_O_15_ and BaCO_3_ in NaCl and KCl molten salts at 950 °C for 3 h. Bi^3+^ in BaBi_4_Ti_4_O_15_ was substituted by the Ba^2+^ from BaCO_3_, yielding BaTiO_3_ template and Bi_2_O_3_ by-product. The Bi_2_O_3_ by-product was removed by diluted nitric acid. To fabricate textured ceramics, PMN-PZT matrix powders with an organic binder (Ferro 73225, Vista, CA) and toluene/ethanol solvents were mixed by ball-milling to prepare ceramic slurries. Next, various contents of templates were added into the slurries under magnetic stirring and the slurries were subsequently casted at the rate of 40 cm min^−1^ by using a doctor blade with a height of 200 µm. The dried green tapes were cut, stacked, and laminated at 75 °C under 20 MPa pressure for 15 min. The green samples were heated to 400 °C with a heating rate of 0.3 °C min^−1^ and held for 2 h to remove organic solvent and binder, and then cold isostatically pressed under 133 MPa for 1 min. Samples were subsequently sintered at 1150 °C for 10 h in flowing O_2_ (0.2 L min^−1^). For comparison, PMN-PZT random ceramics were prepared by the same processing as textured PMN-PZT ceramic but without adding BaTiO_3_ templates. The microstructures of BaTiO_3_ template, PMN-PZT random ceramics, and PMN-PZT textured ceramics were observed by scanning electron microscopy (ESEM Q250, FEI, Netherlands) are shown in Supplementary Fig. 9. More details about the synthesis of BaTiO_3_ template and textured PMN-PZT ceramics can be found in elsewhere^13,31^.

### Microstructure characterization

The crystallographic phases of ceramics were characterized using X-ray diffraction (XRD, PANalytical Empyrean, Netherlands). The degree of pseudo-cubic [001] texture was determined from the XRD pattern in the 2 theta range of 20–60^o^ by the Lotgering factor method^32^. The dielectric properties of poled samples as a function of temperature were measured by using a multi-frequency LCR meter (E4980AL, Keysight, USA). The piezoelectric properties of samples as a function of temperature were obtained by resonance and anti-resonance technique using an impedance/gain phase analyzer (E4990A, Keysight, USA). The piezoelectric coefficient *d*_33_ was measured by a d_33_-meter (YE 2730 A, APC Products, Inc., USA). The polarization vs. electric field hysteresis loops and strain vs. electric field curves were measured using a ferroelectric tester (Precision Premier II, Radiant Technologies, Inc., USA).

### Phase-field simulations

To perform the simulation study of electromechanical coupling in textured PMN-PT ceramics, we adopted the phase-field model of polycrystal ferroelectrics. In this model, the polycrystal grain structure is characterized by the grain rotation matrix field **R**(**r**) and the ferroelectric state is described by the polarization vector field **P**(**r**) whose evolution is characterized by the time-dependent Ginzburg-Landau equation. The modeling details can be found in Supplementary Note 3. The electromechanical coupling factor is obtained by simulating *ε*^*T*^ and *ε*^*S*^, where *ε*^*T*^ is the stress-free dielectric permittivity and *ε*^*S*^ is the permittivity with conserved strain. To obtain the corresponding permittivity, we apply a small electric field Δ*E* in the poling direction and measure the induced polarization change Δ*P*.

### First-principles calculation

Calculations for the perovskite ferroelectrics BaTiO_3_ were performed within the framework of density functional theory (DFT) by using the Vienna Ab initio Simulation Package (VASP)^33–36^. The plane-wave basis projector augmented wave (PAW) method^37,38^ was used in the local-density approximation (LDA)^39^. The conjugate-gradient algorithm is used for structure optimization with the plane-wave cutoff energy of 500 eV and an 8 × 8 × 8 Gamma *k*-points scheme, and all atoms are free to move until convergence with forces between them <10^−3^ eV/Å. The Born effective charge tensor Z^*^ is calculated using the density functional perturbation theory (DFPT)^40^. The electric dipole moment for a unit cell is determined by ${{{{{\bf{p}}}}}}=e\sum {{{{{{\bf{Z}}}}}}}_{\alpha }^{* }\cdot {{{{{{\bf{u}}}}}}}_{\alpha }$, where **u**_α_ is the displacement of atom *α*. The calculated Born effective charges in the cubic phase are: ${Z}_{{Ba}}^{* }=2.76$, ${Z}_{{Ti}}^{* }=7.47$, ${Z}_{O\perp }^{* }=-2.17$, and ${Z}_{O\parallel }^{* }=-5.89$.

## Supplementary information

Supplementary Information

## Data Availability

The data supporting the findings of this study are available upon request from the corresponding authors.
