# Supplementary material for: Near-ideal electromechanical coupling in textured piezoelectric ceramics
Source: Nat Commun. 2022 Jun 22;13:3565. doi: 10.1038/s41467-022-31165-y (PMC9217974; doi:10.1038/s41467-022-31165-y)
Supplement: Supplementary file 1 — Supplementary Information [file 41467_2022_31165_MOESM1_ESM.pdf]

Near-ideal electromechanical coupling in textured piezoelectric ceramics

(Supplementary Information)

Yongke Yan[1,*], Liwei D. Geng[2,*], Hairui Liu[1], Haoyang Leng[1], Xiaotian Li[1], Yu U. Wang[2,*], Shashank Priya[1,*]

[1] Department of Materials Science and Engineering, Pennsylvania State University, University Park, PA 16802, USA.

[2] Department of Materials Science and Engineering, Michigan Technological University, Houghton, MI 49931, USA

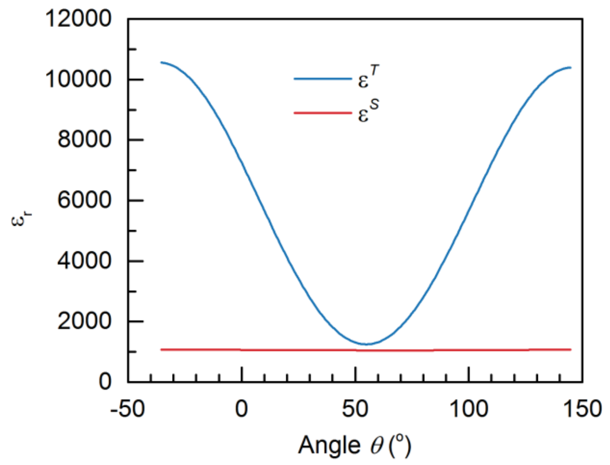

Supplementary Fig. 1 | Phase-field simulated permittivity. Simulated permittivity ε^T and ε^S for the rhombohedral PMN-0.3PT single crystal under electric field with various orientations.

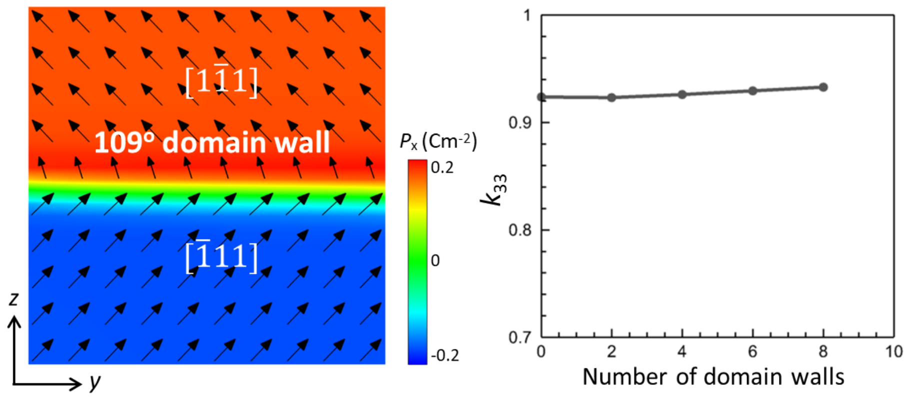

Supplementary Fig. 2 | Domain wall effect on k_{33} determined by phase-field simulation. Simulated electromechanical coupling factor k_{33} as a function of the number of 109° domain walls per micron.

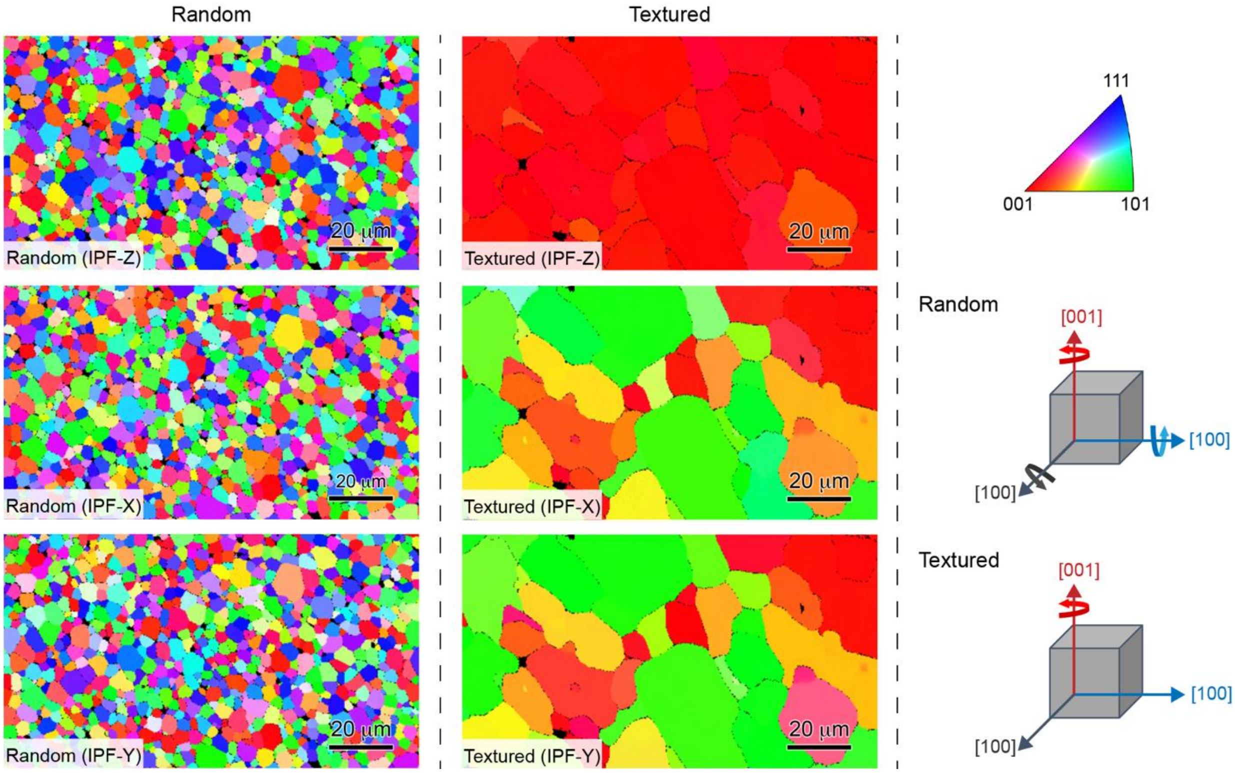

Supplementary Fig. 3 | Grain orientation of random and textured PMN-PZT ceramics. Inverse pole figure (IPF) maps of random and textured ceramics, measured by the SEM-EBSD technique. The IPF component in Z directions reveals the sample's out-of-plane (normal), and the IPF components in X and Y directions reveals the in-plane spatial grain orientation distributions.

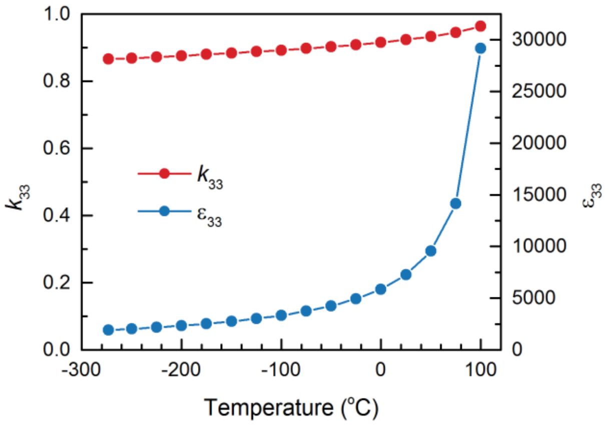

Supplementary Fig. 4 | Temperature effect on k_{33} determined by phase-field simulation. Simulated electromechanical coupling factor k_{33} and permittivity ε_{33}^{T} as a function of temperature up to the phase transition point.

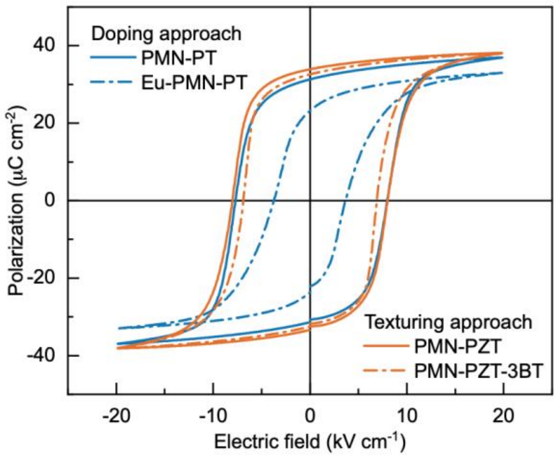

Supplementary Fig. 5 | Comparison of coercive electric field with different approaches. Polarization vs electric field hysteresis loops for PMN-PT, Eu-PMN-PT, PMN-PZT random ceramics and PMN-PZT-3BT textured ceramics. Eu doping dramatically reduces the coercive field E_c of PMN-PT ceramics. On the contrary, texturing has much less impact on the coercive field E_c of PMN-PZT ceramics.

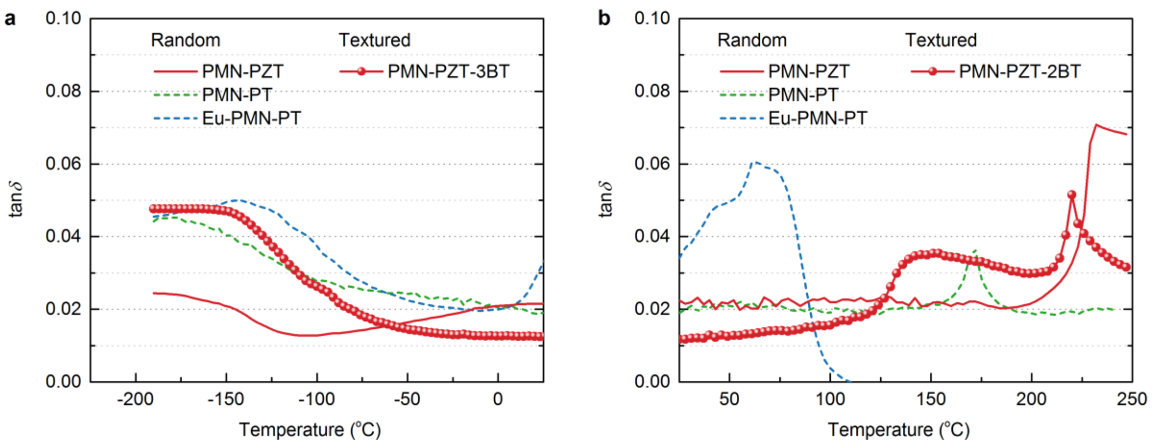

Supplementary Fig. 6 | Temperature dependence of dielectric loss. a, Dielectric loss tanδ measured in low-temperature range from -190 °C to room temperature, **b**, Dielectric loss tanδ measured in high-temperature range from room temperature to 250 °C.

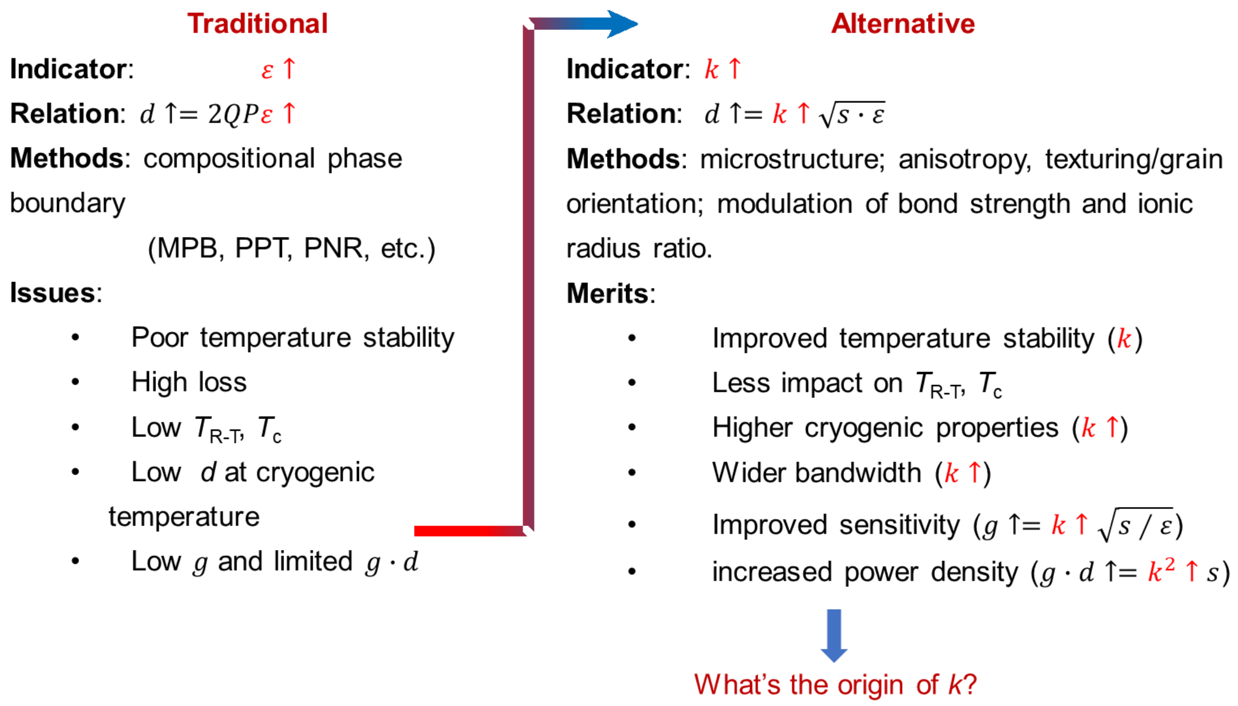

Supplementary Fig. 7 | Pictorial comparison between traditional approach and alternative approach of designing piezoelectric materials proposed in this study.

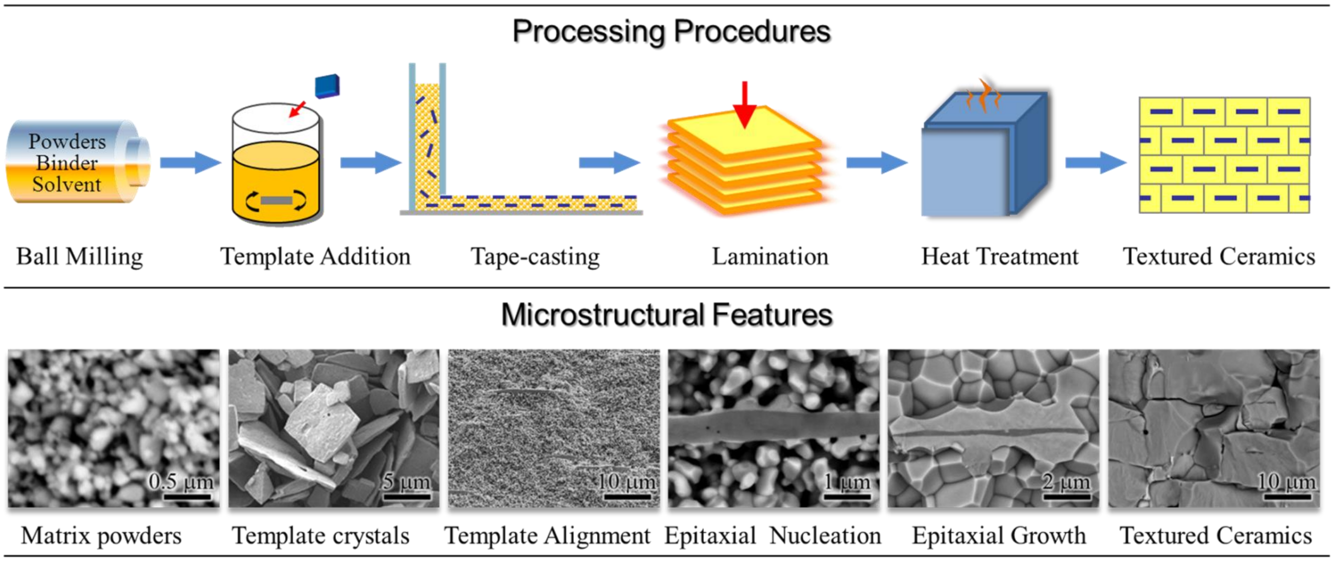

Supplementary Fig. 8 | Schematic illustration of templated grain growth process for synthesizing piezoelectric textured ceramics.

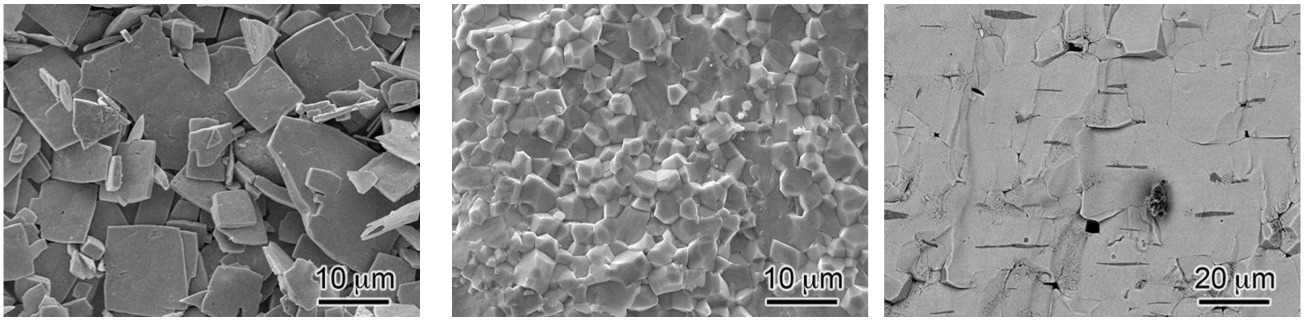

a **b** **c**

Supplementary Fig. 9 | Microstructures. a, SEM image of $BaTiO_3$ template. **b**, SEM image of random PMN-PZT ceramics. **c**, SEM image of 3 *vol*% $BaTiO_3$ template textured PMN-PZT ceramics.

Supplementary Note 1: The references for k_{33} and k_{31} data in Fig. 3e and 3f in main manuscript

(1) Single crystal k_{33} data in Fig.3e:

PMN-PT: [1]; PZN-PT: [2]; PIN-PMN-PT: [3]; PMN-PZT:[4]

(2) Ceramics k_{33} data in Fig.3e:

Sm-PMN-PT: [5]; PZT-5A: [6]; PZT-5H: [6]; PZT-4: [6]; PZT-8: [6]

(3) Single crystal k_{31} data in Fig.3f:

PMN-PT: [1]; PMN-PT(45): [1]; PMN-PZT(45):[4]; PMN-PZT(45):[4]

(4) Ceramics k_{31} data in Fig.3f: PZT-5A: [6]; PZT-5H: [6]; PZT-4: [6]; PZT-8: [6]

Supplementary Note 2: Theoretical model of perovskite ferroelectrics

In this theoretical model of perovskite ferroelectrics, in order to capture the key contributions to ferroelectricity, we consider the total energy that consists of the short-range repulsive energy, the long-range Coulomb energy, and the hybridization covalent bonding energy. Because of the complexity of the exact total energy, we assume that the A-O bond is purely ionic while the B-O bond is a mixture of ionic and covalent bonding, inspired by the bonding nature of $BaTiO_3$. In the tetragonal phase, we neglect all the transverse interactions and only keep the longitudinal interactions. Thus, the simplified total energy that considers the off-centering displacement *r* is formulated as

$$U(R,r) = 2\lambda_A e^{-R/\rho_A} + 2\lambda_B e^{-R/\rho_B}\cosh\left(r/\rho_B\right) - 2\lambda_{Bc} e^{-R/\rho_{Bc}}\cosh\left(r/\rho_{Bc}\right) - \alpha\left(\frac{r}{R}\right)\frac{q^2}{4\pi\varepsilon_0} - Eqr\,, \qquad (4)$$

where the first and second term respectively represent the short-range repulsive energy of A-O and B-O bonds[7], the third term describes the covalent bonding energy of B-O hybridization[8], the fourth term is the long-range Coulomb (Madelung) energy[7], and the last term is the electric field energy. *r* is the ionic displacement of B, which characterizes the polarization. $2R$ is the lattice constant, which is related to the deformation or strain. ρ and λ are empirical parameters, where ρ can reflect the ionic radii and λ represents the bonging strength. In this work, $x \equiv \rho_A/\rho_B$ implies the ionic radius ratio between A and B ions, while $y \equiv \lambda_{Bc}/\lambda_B$ characterizes the relative covalent bonding strength of the B-O bond. The derived Madelung function in the fourth term which depends on the off-centering displacement *r* is given by

$$\alpha\left(\frac{r}{R}\right) = 2\alpha_0 - \left[\psi^0\left(\frac{r}{R}\right) + \psi^0\left(-\frac{r}{R}\right)\right] + \frac{1}{2}\left[\psi^0\left(\frac{r}{2R}\right) + \psi^0\left(-\frac{r}{2R}\right)\right] - \gamma\,, \qquad (5)$$

where $\alpha_0 = 2ln2$ is the Madelung constant, ψ is the Polygamma function, and γ is the Euler-Mascheroni constant.

Supplementary Note 3: Phase-field model of ferroelectric polycrystal

In this ferroelectric polycrystal model, the grain structure of a polycrystal is characterized by a grain rotation matrix field **R**(**r**) that describes the geometry (size, shape, location) and crystallographic orientation (texture) of individual grains. The state of such a ferroelectric polycrystal is described by a polarization vector field **P**(**r**), and the total system free energy under externally applied electric field $\mathbf{E}^{ex}$ is[9-12]

$$F=\int d^3r\left[f\left(R_{ij}P_j\right)+\frac{\beta}{2}\frac{\partial P_i}{\partial r_j}\frac{\partial P_i}{\partial r_j}-E_k^{ex}P_k\right]+\frac{1}{2}\int\frac{d^3k}{(2\pi)^3}\left[\frac{n_in_j}{\varepsilon_0}\tilde{P}_i\tilde{P}_j^*+K_{ijkl}\tilde{\varepsilon}_{ij}^0\tilde{\varepsilon}_{kl}^{0*}\right], \tag{1}$$

where

$$\begin{aligned} f(\mathbf{P}) &=\alpha_1\left(P_1^2+P_2^2+P_3^2\right)+\alpha_{11}\left(P_1^4+P_2^4+P_3^4\right)+\alpha_{12}\left(P_1^2P_2^2+P_2^2P_3^2+P_3^2P_1^2\right)\\ &+\alpha_{111}\left(P_1^6+P_2^6+P_3^6\right)+\alpha_{112}\left[P_1^4\left(P_2^2+P_3^2\right)+P_2^4\left(P_3^2+P_1^2\right)+P_3^4\left(P_1^2+P_2^2\right)\right]\\ &+\alpha_{123}P_1^2P_2^2P_3^2 \end{aligned} \tag{2}$$

is Landau-Ginzburg-Devonshire free energy function of ferroelectric single crystal[13]. It is worth noting that **P**(**r**) in Eq. (1) is defined in a global coordinate system attached to the polycrystal, while **P**(**r**) in Eq. (2) is defined in a local coordinate system aligned with <100> lattice axes of a ferroelectric single crystal, and the operation $R_{ij}P_j$ in $f\left(R_{ij}P_j\right)$ in Eq. (1) transforms **P**(**r**) from the global sample system to the local lattice system in each grain.[16] The gradient term characterizes energy contribution from polarization gradient in domain wall regions, where β is gradient coefficient. The **k**-space integral terms characterize the domain configuration-dependent electrostatic energy of polarization distribution **P**(**r**) and elastostatic energy of misfit strain distribution $\boldsymbol{\varepsilon}^0(\mathbf{r})$, where ε_0 is permittivity of free space, $\tilde{\mathbf{P}}(\mathbf{k})$ and $\tilde{\boldsymbol{\varepsilon}}(\mathbf{k})$ are the Fourier transforms of the respective field variables $\mathbf{P}(\mathbf{r})$ and $\boldsymbol{\varepsilon}(\mathbf{r})$. The spontaneous electrostrictive strain $\boldsymbol{\varepsilon}$ is coupled to the polarization $\mathbf{P}$ through electrostriction coefficient tensor Q_{ijkl}, $\varepsilon_{ij}=Q_{ijkl}P_kP_l$. $K_{ijkl}=C_{ijkl}-n_mC_{ijmn}\Omega_{np}C_{klpq}n_q$, $\Omega_{ik}=(C_{ijkl}n_jn_l)^{-1}$, C_{ijkl} is elastic modulus tensor, and $\mathbf{n}=\mathbf{k}/k$. The spatial-temporal evolution of the polarization $\mathbf{P}(\mathbf{r},t)$ in response to varying electric field $\mathbf{E}^{ex}(t)$ is characterized by the time-dependent Ginzburg-Landau equation[9-12]

$$\frac{\partial\mathbf{P}(\mathbf{r},t)}{\partial t}=-L\frac{\delta F}{\delta\mathbf{P}(\mathbf{r},t)}, \tag{3}$$

where L is kinetic coefficient. In this work, we consider the ferroelectric material PMN-0.3PT, whose material parameters have been experimentally determined[14]: α_1=0.745(T+160.45)×10^5 m/F, α_{11}=-

0.50×10^{8} m^{5}/C^{2}F, α_{12}=-0.5125$\times10^{8}$ m^{5}/C^{2}F, α_{111}=0.5567$\times10^{9}$ m^{9}/C^{4}F, α_{112}=1.333$\times10^{9}$ m^{9}/C^{4}F, α_{123}=0.24$\times10^{9}$ m^{9}/C^{4}F. T is the temperature in kelvin. The elastic modulus constants are C_{11}=275GPa, C_{12}=179GPa, and C_{44}=54.3GPa, while the electrostrictive coefficients are Q_{11}=0.055 m^{4}C^{-2}, Q_{12}=-0.023 m^{4}C^{-2}, and Q_{44}=0.0315 m^{4}C^{-2}.

References

1 Zhang, S. J., Lee, S. M., Kim, D. H., Lee, H. Y. & Shrout, T. R. Elastic, piezoelectric, and dielectric properties of $0.71Pb(Mg_{1/3}Nb_{2/3})O_3$-$0.29PbTiO_3$ crystals obtained by solid-state crystal growth. *J. Am. Ceram. Soc.* **91**, 683-686 (2008).

2 Park, S. E. & Shrout, T. R. Ultrahigh strain and piezoelectric behavior in relaxor based ferroelectric single crystals. *J. Appl. Phys.* **82**, 1804-1811 (1997).

3 Zhang, S. J., Luo, J., Hackenberger, W. & Shrout, T. R. Characterization of $Pb(In_{1/2}Nb_{1/2})O_3$-$Pb(Mg_{1/3}Nb_{2/3})O_3$-$PbTiO_3$ ferroelectric crystal with enhanced phase transition temperatures. *J. Appl. Phys.* **104**, 064106(2008).

4 Zhang, S. J., Lee, S. M., Kim, D. H., Lee, H. Y. & Shrout, T. R. Temperature dependence of the dielectric, piezoelectric, and elastic constants for $Pb(Mg_{1/3}Nb_{2/3})O_3$-$PbZrO_3$-$PbTiO_3$ piezocrystals. *J. Appl. Phys.* **102**, 114103 (2007).

5 Li, F. *et al.* Ultrahigh piezoelectricity in ferroelectric ceramics by design. *Nat. Mater.* **17**, 349-354 (2018).

6 Inc., E. Electro-Ceramic Products and Material Specification. (2011).

7 Kittel, C. *Introduction to Solid State Physics*. (Wiley, 2004).

8 Morse, P. M. Diatomic Molecules According to the Wave Mechanics. II. Vibrational Levels. *Phys. Rev.* **34**, 57-64 (1929).

9 Yang, W. & Chen, L.-Q. Computer Simulation of the Dynamics of 180° Ferroelectric Domains. *J. Am. Ceram. Soc.* **78**, 2554-2556 (1995).

10 Hu, H.-L. & Chen, L.-Q. Three-Dimensional Computer Simulation of Ferroelectric Domain Formation. . *J. Am. Ceram. Soc.* **81**, 492-500 (1998).

11 Semenovskaya, S. & Khachaturyan, A. G. Development of ferroelectric mixed states in a random field of static defects. *J. Appl. Phys.* **83**, 5125-5136 (1998).

12 Wang, Y. U. Field-induced inter-ferroelectric phase transformations and domain mechanisms in high-strain piezoelectric materials: insights from phase field modeling and simulation. *J. Mater. Sci.* **44**, 5225-5234 (2009).

13 Devonshire, A. F. XCVI. Theory of barium titanate. *The London, Edinburgh, and Dublin Philosophical Magazine and Journal of Science* **40**, 1040-1063 (1949).

14 Zhang, H. *et al.* Phase coexistence and Landau expansion parameters for a $0.70Pb(Mg_{1/3}Nb_{2/3})O_3$-$0.30PbTiO_3$ single crystal. *Phys. Rev. B* **96**, 054109 (2017).
